# Cultural impediments to frank communication regarding end-of-life care between older nursing home residents and their family members in Taiwan: a qualitative study

**DOI:** 10.1186/s12912-022-01143-2

**Published:** 2022-12-14

**Authors:** Hsin-Tzu Sophie Lee, Chia-Ling Yang, Sei-Ven Leu, Wen-Yu Hu

**Affiliations:** 1 Department of Nursing, Tzu Chi University of Science and Technology, Hualien City, 97005 Taiwan, Republic of China; 2 Mackay Junior College of Medicine, Nursing and Management, Taipei City, 11260 Taiwan, Republic of China; 3 Department of Computer Center, Tzu Chi University, Hualien City, 97071 Hualien County Taiwan, Republic of China; 4 Department of Nursing, National Taiwan University, Taipei City, Taiwan, Republic of China

**Keywords:** Culture, End-of-life, Nursing home, Advance care planning, Advance directives, Making decisions

## Abstract

**Background:**

When older nursing home residents and their families are faced with end-of-life care decisions in Taiwan, they make them in the context of traditional cultural norms and socioeconomic changes. Both parties (residents and their family members) are often unwilling to broach the topic, leading to a decisional impasse. The aim of this study was to understand difficult-to-raise issues related to end-of-life care by investigating the perspectives of older nursing home residents and their family members.

**Methods:**

This qualitative descriptive study was conducted using content analysis based on the Consolidated Criteria for Reporting Qualitative Research. Purposive sampling was used to select the participants, and sampling continued until data saturation. Data were collected using semi structured interviews, and related analyses were conducted using an inductive approach.

**Results:**

Ten residents and twelve family members were interviewed individually. Six main themes were identified: (1) the inevitability of a goodbye; (2) a good death; (3) going with or against traditional culture; (4) better a good death than a bad life; (5) abiding by the residents’ decisions; and (6) being willing but unable to take care of residents.

**Conclusion:**

Nursing home residents and their family members’ thoughts on end-of-life care shifted toward the concept of a good death, and they even regarded death as a form of liberation. Health care providers may serve as mediators to counsel a resident and their family members separately, enabling them to speak up and understand each other’s thoughts on end-of-life care before a decision is made so that neither party has regrets.

## Background

According to the Social and Family Affairs Administration of the Ministry of Health and Welfare in Taiwan, the monthly cost of living in a nursing home (NH) can range from US$690 to US$2069 [1]. Most NHs in Taiwan are intended for long-term care, and NH residents tend to be individuals who are older, with cognitive impairment, and with a disability impairing their activities of daily living [2]. NH residents thus tend to have physical and mental comorbidities and often experience repeat hospitalisation. Therefore, concerns related to end-of-life (EOL) care are particularly relevant to Taiwanese NH residents [3], and to ensure preserve their autonomy at end of life, older residents should make use of advance directives (ADs) [4].

In Taiwan, ADs include three types of documents: a do-not-resuscitate (DNR) directive, a healthcare proxy decision-maker, and living wills. Among these documents, DNR is one of the most commonly discussed ADs in NHs [3, 5]. However, residents and their family members rarely discuss ADs with each other, which has resulted in Taiwanese NHs having a low DNR directive completion rate (9–20%) [3, 6–9]. A study of 29 older residents living in a NH, who received an intervention in the form of an individual interview based on an advanced care planning (ACP) handbook and a group patient education programme, reported that only six participants signed a DNR directive after the intervention [9]. In addition, according to the results of Tsai et al., the mean interval between NH admission and the signing of a DNR directive was 2.3 years, and whether a resident had completed a DNR directive was related to whether they had a nasogastric tube and the number of times they had been transferred to the hospital. This was because these events led to the hospital physician discussing the residents’ EOL care with family members. In such cases, the family members collectively made EOL care decisions and signed DNR directives for the residents [3].

In Taiwan, family members generally take responsibility for medical decision-making for patients even if the patient is lucid [10]. According to Cheung et al. [11] and Lee et al. [12], in traditional Chinese culture, EOL care decisions tend to be made by the family as a group on behalf of an older family member. In such decision-making, power asymmetries may be present and influenced by the family member’s financial capacity, education level, age, and gender; in particular, gender can be particularly influential because traditional Chinese culture tends to be patriarchal. A study conducted by Tsai et al. [3] reported a 12.97% completion rate (*n* = 73/563) of DNR directives among six NHs, and most of these directives were signed by residents’ family members (90.1%). These findings have proved that cultural concerns may hinder AD completion [3, 7, 13]. Specifically, Chinese cultural concerns about familism [5, 6, 14], the avoidance of talk about death [3, 9, 15], and filial piety [5, 10, 15, 16] greatly hinder conversations about EOL care in Chinese families.

Westernisation and economic development in Taiwan have resulted in a greater emphasis on autonomy (specifically, the autonomy of the older adult to make decisions about their EOL care) over the traditional Chinese values of filial piety and familism [17]. Lee et al. [12] reported that among family members of lucid residents, half of them (*n* = 6/12) expressed hope that the older adult resident would choose their form of EOL care. These family members refused, for fear of broaching the topic, to make EOL care decisions for the resident in advance. However, because family members do not initiate such conversations, residents also tend to leave EOL care decisions to their family members despite having a preference [5, 13]. This unwillingness to broach the subject of EOL care by both parties leads to a decisional impasse.

This study thus aimed to understand difficult-to-raise issues related to EOL care by investigating the perspectives of both older NH residents and their family members.

## Methods

### Study design

This was a qualitative descriptive study that used semi-structured, individual, face-to-face interviews with Chinese older residents living in a NH and with the family members of older NH residents. A qualitative, descriptive study design can be particularly useful for understanding participants’ views on sensitive topics. This study adopted a qualitative, descriptive approach to investigate difficult-to-raise issues related to EOL care from the perspectives of older NH residents and their family members. Semi-structured, individual, and face-to-face interviews were used to collect data because such interviews have been reported to be the most suitable and commonly used approach to data collection in studies with qualitative, descriptive designs [18, 19]. A 32-item checklist was also employed in accordance with the Consolidated Criteria for Reporting Qualitative Research (COREQ) [20].

### Participants and sampling

NH residents aged 65 years or older and their family members aged 18 years or older were eligible to participate. Individuals who had obvious cognitive impairment or were unable to speak Mandarin or Taiwanese were excluded. Purposive sampling was adopted, and participants were recruited through referrals by nurses working in the NH. The first author asked the prospective participants whether they would be willing to participate in the study. Participants were sampled until content saturation was reached. The authors adopted the model of inductive thematic saturation, which indicates that further data collection does not generate new insight once no additional meaningful units are generated [21, 22].

### Data collection and analysis

Before the study began and the face-to-face interviews were conducted, the first author developed two sets of interview guides (one for the interviews with the older residents and another for those with the residents’ relatives) and discussed the questions with experienced EOL care workers and the fourth author. The in-depth, face-to-face interviews were conducted for 30–40 minutes in the NH, and they involved open-ended questions (Table 1). All participants were asked the same questions, and all interviews were conducted by the first author in Mandarin or Taiwanese. All interviews were audio recorded for accurate data transcription.

**Table 1:** Interview guide for older residents and their families

Residents 1. How do you feel about your present health condition compared with others or compared with how it was last year? 2. What will you worry about when you are approaching the end of life? 3. If your condition becomes severe, what type of care or treatment would you choose? How will you talk to your family members about this choice? 4. In what kind of situation would you prefer to talk about issues related to end-of-life treatment or care with your family members? Why? 5. What are your opinions with regard to signing advance directives for yourself? 6. In terms of end-of-life care, what do you think your family should do with regard to fulfilling their duty of filial piety? 7. What would you like to say to your family members about your end-of-life care preferences but that you think are difficult to raise during discussions with them?	
Residents’ relatives 1. If the resident were at the end of life, how would you feel? 2. If the condition of the resident worsens, what type of care or treatment would you want them to have? 3. In what kind of situation would you prefer to talk about issues related to end-of-life treatment or care with the resident? Why? 4. Do your family members or the resident not like to talk about issues related to end-of-life care with each other? Why? 5. What do you think about making the end-of-life care decisions in advance for the resident? 6. What are your opinions about signing advance directives for the resident? 7. If the resident tells your family that they want to sign their own advance directives, how would you feel? Would you respect their decision? Why? 8. What things do you want to say to the resident about end-of-life care choices but you think are difficult to raise in discussions with them?	

A qualitative inductive content analysis method was used per the guidelines of Vaismoradi et al. [23] The data were analysed according to the following steps. First, the interview transcripts, field notes, and reflection logs for each participant were recorded in writing and read numerous times by the first author to understand the content. Second, the first three co-authors independently conducted line-by-line coding of a series of transcripts and notes to identify categories, and these authors met with the fourth author to decide on the analytical direction of the study based on the research question. Third, the condensed meaning units were compared by all authors and organised into subthemes; these subthemes were then presented to the participants and revised according to their opinions. Fourth, the revised subthemes were extracted from the remaining reviews and allocated into themes until no new information could be identified from the data, indicating that data saturation was reached. Potential sets of meaning units, subthemes, and themes were separately reviewed by each author to ensure that the study results would be valid. Fifth, the two data sets (those obtained from the older residents and from their family members) were analysed together and were presented as a whole. Finally, after discussion, all authors reached a consensus regarding the potential set of meaning units, condensed meaning units, subthemes, and themes.

### Trustworthiness

Because the trustworthiness must be ensured in qualitative research, member checking, an audit trail, and a reflexive journal were used to ensure the research data’s credibility, dependability, and confirmability, respectively [24]. First, to implement member checking, each participant was provided with a transcript of their interview and a summary of the extracted subthemes and was asked to determine whether they resonated with the subthemes. Member checking was valuable in this study because the study aim was to understand difficult-to-raise issues related to EOL care by investigating the perspectives of both older NH residents and their family members. The data were returned to the participants for them to give feedback on whether the data were consonant with their experiences. For the audit trail, the first author accounted for all research decisions and activities to reveal how data were collected, recorded, and analysed. The following data were used for the audit trail: raw data, interview and observational notes, records of rationales for decisions, and memos or notes related to data collection and analysis. Finally, to establish data confirmability, a reflexive journal was written by the first author such that they could reflect on, tentatively interpret, and plan data collection. The aforementioned documents were retained for cross-checking during the inquiry process and for writing a final report on this study.

### Ethical considerations

This study was approved by the Medical Foundation Research Ethics Committee of Hualien Tzu Chi hospital. All participants were fully informed of the study’s aim and the methods used to gather data before an interview was conducted. Written informed content was obtained from those who agreed to participate, and those who did not wish to continue participating were free to withdraw at any time. Specifically, the participants signed statements regarding their consent to participate under an ‘Ethics, Consent, and Permissions heading and under a ‘Consent to Publish’ heading; thus, the author obtained consent from the participants to publish unidentifiable individual data.

## Results

Two data sets were obtained: those from the residents and those from the family members. The resident data set comprised data obtained from 10 residents (coded A–J; two men, eight women) aged between 65 and 90 years, with an average age of 80 years. The family member data set comprised data obtained from 12 relatives of the residents (coded AF–JF; four men, eight women). Most were the children or grandchildren (*n* = 10, 83%) of the residents (Table 2). The interviewed family members were the relatives of the 10 interviewed residents. For two residents (coded F and J), two of their family members participated in the study. One resident’s (coded F) two adult children (coded FF-1,2), who were siblings, and another’s (coded J) brother and sister-in-law (coded J-1,2), who were husband and wife, participated in this study. The total number of interviews was 22. The mean duration of the resident interviews was 30 minutes (range 20–40 minutes), and the mean duration of the family member interviews was 36 minutes (range 30–40).

**Table 2:** Characteristics of participating older residents and their family members

Characteristics	Older residents (***n*** = 10)	Residents’ relatives (***n*** = 12)
	Number (%)	Number (%)
Sex:		
Male	2 (20)	4 (33)
Female	8 (80)	8 (67)
Age:		
20–59 years		5 (42)
60–69 years	3 (30)	6 (50)
70–79 years	3 (30)	1 (8)
80–89 years	2 (20)	
> 90 years	2 (20)	
Education status:		
College	1 (10)	8 (67)
Elementary school	7 (70)	4 (33)
No literacy	2 (20)	0 (0)
Religious affiliation		
Buddhist	6 (60)	8 (67)
Taoist	4 (40)	3 (25)
Catholic	0 (0)	1 (8)
MMSE score:		
28–30	3 (30)	10 (83)
27–24	7 (70)	2 (17)
< 24	0 (0)	0 (0)
Relationship of primary caregiver to resident:		
Child or grandchild (son/daughter/grandchild)		10(83)
Sibling (brother/sister)		2 (17)
Time at the long-term care facility:		
1–5 years	6 (60)	
> 5 years	4 (40)	

Content analysis was employed to explore EOL care issues that were difficult to raise in discussions among the residents and their family members (Table 3). Six themes were explored. The first three themes were related to the wishes of residents and their families to give their opinion. The fourth theme was related to residents’ wish to express their opinion, and the fifth and sixth themes were related to family members’ wishes to express their opinion.

**Table 3:** Issues of concern for residents and their families during advance care planning implementation

Classification	Older residents			Residents’ relatives		
	Themes	Subthemes	Categories	Themes	Subthemes	Categories
**Same**	1. The inevitability of a goodbye	1-1 Letting go of life	1-1-1 Dying of old age as natural	1. The inevitability of a goodbye	1-1 Facing the limits of how long one can live	1-1-1 Life has an end
			1-1-2 Death is a natural phenomenon			1-1-2 Death must be faced
		1-2 Being put out of one’s misery	1-2-1 Life brought back by emergency resuscitation only adds to suffering		1-2 The inevitability of death	1-2-1 Life brought back by emergency resuscitation still must end eventually
			1-2-2 Death is liberation			1-2-2 Death is liberation
	2. A good death	2-1 Not needing anything	2-1-1 Refusing to receive emergency care	2. A good death	2-1 Not being given painful life-sustaining treatment	2-1-1 Refusing to receive emergency care
			2-1-2 Avoiding painful treatments			2-1-2 Avoiding painful treatment
		2-2 Wanting to depart without pain	2-2-1 Dying smoothly		2-2 Dying naturally and comfortably	2-2-1 Dying smoothly
			2-2-2 Dying peacefully and comfortably			2-2-2 Dying peacefully and comfortably
	3. Going with or against traditional culture	3-1 Bound by traditional cultural beliefs	3-1-1 Giving up emergency care is considered filial piety	3. Going with or against traditional culture	3-1 Not giving up the chance to live	3-1-1 Trying your best before deciding on the next step
			3-1-2 Family harmony leads to prosperity			
			3-1-3 Fate and reincarceration			
		3-2 Doing good and doing no harm	3-2-1 Avoiding negative end-of-life situations that other older family members experienced		3-2 Not the right moment to make a decision	3-2-1 Previous experiences in handling family members’ medical treatment
**Different**	4. Better a good death than a bad life	4-1 No lingering desire for life	4-1-1 Fear of the negative effects of prolonging life	5. Abiding by the residents’ decisions	5-1 Respecting the resident’s wishes for a good death	5-1-1 Respecting the resident’s desire to receive no emergency care
		4-2 Regretting the family’s decision back then	4-2-1 It would have been nice to go	6. Being willing but unable to take care of residents	6-1 Economic pressure	6-1-1 Economic burden
						6-1-2 Consumption of government resources
					6-2 Failure to fulfil one’s family responsibilities	6-2-1 The burden of raising the next generation
						6-2-2 There is no need for filial piety toward someone who has been sick for a long time

### Theme 1: the inevitability of a goodbye

Residents reported wanting to tell their family that they wished to let go of life. In addition, the residents reported that they believed that death by old age was natural, that being saved through the effort of emergency care only adds to suffering, and that death is similar to being put out of their misery:

People come and go and are born to die, sooner or later … emergency resuscitation does not set people free. It makes people lie in bed in pain, just like where I am right now. I don’t want emergency care; I want to be relieved. (I).

Most family members wanted to tell the residents that they believed saying goodbye to be inevitable. They brought up thoughts about facing the limits of how long one can live and the inevitability of death. Family members mentioned that life has an end and that death must be faced:

Life has a time limit... When you are old, you can only live for a few more years, even after receiving emergency care... The death of an elderly family member is something that each person must face. (FF-1).

Half of the family members reported that a life brought back through emergency resuscitation still must end eventually and that death is liberation:

If a person can only lie in bed after receiving emergency care, it is a matter of time until they will be gone for the last time... let them pass with dignity—it will be a relief for them and all of us. (JF-2).

### Theme 2: a good death

All residents stated that they wanted to tell their families about their preference for a good death in case their condition worsens. They mentioned not needing anything and wanting to depart without pain:

When that day comes, I can decide for myself. I won’t need anything... like this (using the index finger to point to the picture of intubation) and that (using the index finger to point to the picture of cardiac massage) or this (using the index finger to point to the picture of electric shock therapy) are not needed. Let me pass away smoothly and peacefully. (C).

Most family members also stated they wanted to tell the residents that they wished they would have a good death. They brought up thoughts of not being given painful life-sustaining treatment and dying naturally and comfortably:

We hope she doesn’t need to receive aggressive treatment, including endotracheal intubation, cardiac massage, electric shock—don’t make her suffer anymore … we will consider hospice care. (CF).

### Theme 3: going with or against traditional culture

With regard to EOL care, residents noted being bound by traditional cultural beliefs, such as Confucianism (filial piety and the idea that familial harmony leads to prosperity) and Buddhism (karma and reincarnation) and wanting to tell their family members that it was up to families to decide:

I wanted to tell my children that I don’t want to be saved...But if they want me to be saved, so be it. I want to make it easier on them, and I believe in human reincarnation...I accept whatever my children do, and that’s my destiny. (F).

However, residents also held the ethical belief of doing good and doing no harm. Because of this, the three residents were anxious to tell their family members that they need not adhere to traditional norms if doing so leads to harm:

The ancients said that if something happens to parents and children give up without trying to save their parents, those children are considered unfilial. I disagree. Since I was saved, I’ve been lying in bed the whole time. It is very painful. I want to tell them to let me pass away with dignity, which is what I think filial piety should be. (A).

Two family members, however, noted being bound by traditional norms, and they thus adhered to the attitude of that one should not give up the chance to live and believed that it was not the right moment to make a decision. The family members who insisted on not giving up the chance for them to live believed that they needed to do everything possible to sustain life before the next step could be decided:

If my mother is seriously ill, I have to do my best … to keep her alive as long as possible because medical technology is advancing. (FF-2).

The beliefs of family members who stated that there was no need to decide now may have been influenced by past medical experiences:

I regretted that my dad passed away without receiving good medical treatment at the time. I don’t want the same thing to happen to my mother...I must try my best to save her, and I don’t have to decide on EOL care right now. (GF).

### Theme 4: better a good death than a bad life

Residents expressed their desire to tell their families, better a good death than a bad life. They mentioned that they have no lingering desire for life and regret the family’s decision back then. The remark about lingering desires was associated with residents’ fear of the negative effects of prolonging life:

Receiving emergency treatment is just a continuation of pain. Even if the person’s life is saved, being half-dead is even more painful. (B).

Their regrets stemmed from their fear and sorrow associated with previous emergency care experiences. They stated that it would have been nice to go:

The last year my condition was not good. It would be great if I just passed away...I was saved last time—I am now just lying here day by day and counting down to my final days...It’s very painful [the interviewee cries]. (H).

### Theme 5: abiding by the residents’ decisions

Most family members stated that they would respect the resident’s wishes if they knew in advance that the resident wanted to have a good death and did not want emergency treatment.

If my mother says that she has a preference for a good death or for hospice care, I will certainly respect that and will do what she wants. (EF).

### Theme 6: being willing but unable to take care of residents

Regarding the EOL care of residents, the family members expressed a sense of being willing but unable to take care of residents to do so due to economic pressure and a failure to fulfil their family responsibilities. Economic pressure was indicated by mentions of economic burden and consumption of government resources:

If there was no government subsidy, taking care of my brother would have cost me everything a long time ago. Sometimes I do not want to pay attention to him, but when I think that he is my brother, I grit my teeth and keep going…I don’t know how long it will be like this. (JF-1).

In terms of failing to fulfil responsibilities, three family members mentioned the burden of raising the next generation and indicated that there is no need for filial piety toward someone who has been sick for a long time:

We as family members certainly don’t want our relatives to leave. We want to keep them by our side. However, no filial son can be by the bedside of a parent who has been sick for a long time.... I have children to support, so I have to make a choice... (IF).

## Discussion

Discussion and decision-making regarding EOL care are difficult for residents and their family members [4]. The results of this study indicated that when faced with decisions about EOL care, both residents and relatives wanted to talk to each other about the inevitability of a goodbye, the preference for a good death, and the question of going with or against traditional culture. The residents wanted to tell their family members to let go because death is inevitable. This finding is consistent with those of previous studies reporting that older people tended to believe that people are born to die [6] and have a positive attitude toward death [5, 15]. Family members wanted to say that they know that the resident’s time on Earth is limited. Previous studies have proposed a similar point: most family members of NH residents believe that the resident will be gone eventually [8, 12]. The residents would rather die than be made to live out the remaining time of their lives suffering due to receiving emergency care. Family members also admitted that the death of the resident, despite being painful, would be a relief for everyone. This finding of both parties viewing death as liberation from suffering is less reported in the literature.

With regard to going with or against traditional culture, the residents were conscious of the traditional Confucian belief that familial harmony leads to prosperity and the Buddhist concepts of fate and karma. In Chinese cultural, discussions regarding EOL care are considered taboo and may disrupt familial harmony [5, 6, 10, 25]. The residents who participated in this study believed that talking about EOL care would cause their family members to feel upset and therefore may be troubling for the family members. Thus, residents chose to leave EOL decisions to their family. They believed that doing so would maintain familial harmony and make it easier for their family members. A study [6] indicated that the success of ACP promotion in Chinese societies depends on the Confucian value of harmony in general and familial harmony in particular. In Chinese culture, familial relationships are characterised by harmonious interdependence [25]. The results of the present study corroborate previous findings. The residents reported believing that discussing EOL care would disrupt familial harmony and that they offer the right to make EOL decisions to their family members to avoid disputes. The residents were also inclined to accept the burdens resulting from emergency care if the decision to administer it is made by their family because that would be their karma and destiny. This finding agrees with those of previous studies indicating that the Buddhist concepts of karma, reincarnation, and destiny have deep roots in Chinese culture [5, 9, 16]. Although the older residents in this study were influenced by traditional norms, they wanted to tell their family members that letting them die (without giving them emergency care) would constitute true filial piety. This indicates that the residents were inclined to go against the cultural norm of doing everything within one’s power to prolong life. This may be because the residents witnessed their parents experiencing a negative EOL as a result of the emergency care they received, and this may have influenced their preferences for their own care [15].

Unlike the residents, the family members adhered to the life-preserving demands of filial piety before they learned about the residents’ views on EOL care. Thus, the family members believed that EOL care decisions could wait. This finding is also consistent with the findings of previous research [8, 12, 17]. The withdrawal of treatment may result in a heavy moral burden for family members due to the generational gap between them and the resident. The Chinese conception of filial piety is such that children must do all they can to preserve their parents’ lives [6, 12, 17]. Interestingly, most family members (*n* = 10) in this study stated that if they knew that the residents wanted to opt out of emergency care, they would respect their wish to do so. This finding corroborates those of previous studies and indicates that such family members often respect the autonomy of the older adult to choose their EOL care, possibly due to increasing Westernisation and economic development [12, 17].

This study presents two points that have been rarely addressed in previous research. First, more than half of the residents wanted to tell their families that the ancient quote ‘better a bad life than a good death’ should be changed to ‘better a good death than a bad life’. Five residents who had been resuscitated were later placed in long-term care institutions. They indicated that their family members’ decision to let them receive emergency care was wrong and that they wished to have passed away instead. Second, a quarter of the family members expressed a reluctance to talk about raising grandchildren in addition to taking care of their parents. They intended to continue to look after the resident but had limited resources and energy. Some family members even said that they were waiting for the resident to go. It thus appears that both the residents and their family members were waiting for death to come as a relief. In other words, they both understood that death is unavoidable and discussed the desire for a good death; however, they refrained from sharing their perspectives due to cultural beliefs about familial harmony, fate, karma, and filial piety. Without knowing that the resident’s wish for a good death aligns with theirs, family members simply fall back on the concept of life preservation related to filial piety during times of emergency.

Although Westernisation and economic development in Taiwan have resulted in an emphasis on self-determination over the traditional Chinese values of filial piety and familism, which have led many family members believe that NH residents’ autonomy and right to make decisions related to their EOL care must be respected [7, 12]. In Chinese culture, a taboo toward discussions of death [5, 6, 10], the value of filial piety [6, 10, 16], and a belief that familial harmony leads to prosperity [26] have led NH residents and family members to be reluctant to discuss EOL care with each other. This unwillingness to broach the topic of EOL care by both parties leads to a decisional impasse. Under such conditions, to engage residents and family members in a discussion of EOL wishes in a manner that honors the cultural emphasis on filial piety and familial harmony, first, healthcare providers should conduct interviews with residents to understand their feelings regarding living in an NH and their thoughts regarding the future and EOL care. If residents broach the topic of a good death, healthcare providers can use AD documents to explain and determine residents’ views on EOL care. After, the healthcare providers can interview the residents’ family members and discuss the residents’ choices regarding their EOL care and assist both parties in reaching a consensus on the residents’ EOL care.

## Conclusion

Taiwanese society has become increasingly Westernised, and older adults and their family members wish to communicate their thoughts on EOL care [12, 17]. However, discussions about death are regarded as taboo by Chinese people [5, 6, 10]. Chinese cultural values of filial piety [6, 10, 16] and the idea that familial harmony leads to prosperity [27] also informs discussions on EOL care. In particular, both parties refrain from discussing topics related to death for the sake of preserving harmony. Many Chinese people avoid discussing contentious or sensitive issues to preserve a sense of harmony. Under the influence of the idea that familial harmony leads to prosperity, most Chinese families appear happy and harmonious. However, this harmony can be an illusion based on ‘forbearance for the sake of family’ (相忍為家 in Chinese). Situations where decisions are the result of compromise are crucial in ACP, and the negotiation required to reach this compromise is difficult to realise in Chinese society [26, 27]. When long-term care facilities promote ACP, healthcare providers can act as mediators and separately counsel residents and family members. This can enable them to express their opinions and to understand each other’s thoughts regarding EOL care before making decisions to ensure that neither party regrets the decisions. Residents will have fewer regrets because they may wish to experience a good death without disrupting familial harmony, and family members will have fewer regrets because they will be able to follow the residents’ decisions regarding their EOL care without feeling guilty about having failed to discharge their filial duties.

### Strengths and limitations

The strengths of this study are its rigorous methodological approach and recruitment of participants who were older residents and their family members. In addition, the present study contributes to the literature by exploring the similarities and differences in the beliefs of older residents and their family members regarding discussing EOL care. Research on ACP implementation in long-term care facilities in Taiwan is generally lacking. Therefore, the results of the present study offer novel insight into perceptions of EOL care in Taiwan and contribute to the literature and evidence base regarding the subject. The present study has the limitation of NH staff not being recruited as participants despite such staff playing a crucial role in providing care for residents. Further research is required to explore the perspectives of NH staff on discussions related EOL care between older NH residents and their family members.

## Data Availability

The dataset supporting the conclusions of this article is included within the article. However, the raw dataset cannot be shared publicly due to sensitive nature of the questions asked in the study. The data are available from the corresponding author upon reasonable request.

## Abbreviations

EOL: End-of-Life
COREQ: Consolidated Criteria for Reporting Qualitative Research
ACP: Advance Care Planning
ADs: Advance Directives
DNR: Do Not Resuscitate
